# The plasma fibrinogen levels in the nitroglycerin-induced chronic migraine rat model and its association between migraine-associated vestibular dysfunction

**DOI:** 10.3389/fneur.2023.980543

**Published:** 2023-03-24

**Authors:** Jilei Zhang, Yixin Zhao, Yuanyuan Jing, Lin Han, Xin Ma, Lisheng Yu, Tongxiang Diao

**Affiliations:** Department of Otolaryngology–Head and Neck Surgery, Peking University People’s Hospital, Beijing, China

**Keywords:** migraine, plasma fibrinogen, vestibular dysfunction, hyperalgesia, nitroglycerin

## Abstract

The purpose of this study was to measure the vestibular function and plasma fibrinogen level in the nitroglycerin (NTG)-induced chronic migraine rats, and explore the effect of defibrinogenation on migraine and associated vestibular dysfunction. The chronic migraine rat model was built by recurrent NTG injection. Batroxobin was administrated as a defibrinogenating drug. We measured the mechanical withdrawal threshold, vestibular function, and fibrinogen level of the rats 30 min before and 2 h following the model establishment, as well as 1 h after batroxobin administration. The results showed that vestibular function was impaired in NTG-induced chronic migraine rats. The fibrinogen levels were increased following repeated NTG injections. However, defibrinogenation did not affect either aggravating or alleviating mechanical hyperalgesia or vestibular dysfunction in the migraine model rats. These findings suggest that the NTG-induced chronic migraine rat model can be used for research on migraine-associated vestibular symptoms. Albeit the association between elevated fibrinogen levels and migraine attacks can be observed, the role of excessive fibrinogen in the pathogenesis of chronic migraine is yet to be determined.

## 1. Introduction

Migraine is a chronic episodic neurological disorder characterized by moderate or severe headache attacks and neurological symptoms (1). It is estimated that 1.04 billion people suffer from migraine worldwide, with an age-standardized prevalence of 14.4% (2). Migraine is a highly disabling disease, not only seriously affecting patients’ quality of life, but also imposing a huge socioeconomic burden (3). Vestibular symptoms are common in patients with migraine (4), and it can further augment the negative impact on quality of life, which cause growing concern (5, 6). In the appendix of the International Classification of Headache Disorders 3-beta (ICHD-3β), the diagnostic criteria of vestibular migraine were published (7). As a tertiary vertigo center in the department of otolaryngology, patients with vestibular migraine are more common than migraine without vestibular symptoms in our outpatient clinic. However, most of the studies on migraine and vestibular dysfunction are based on clinical observations (8) and lack animal models to explore the mechanism of the association in experiments.

The pathogenesis of migraine remains unclear. Epidemiologic studies have shown that migraine is a risk factor for ischemic stroke, especially in young women without other well-established vascular risk factors (9, 10), which suggests that hypercoagulability may play a role in migraine (11). Fibrinogen is a glycoprotein involved in the final step of the coagulation cascade and is a major determinant of whole blood and plasma viscosity (12). Previous studies have found that the levels of plasma fibrinogen were increased in patients with migraine, particularly migraine with aura (13, 14). In addition, there is also a potential association between the presence of a prothrombotic condition and vestibular dysfunction (15, 16), and increased plasma levels of fibrinogen were observed in patients with acute peripheral vertigo as well (17).

As previously reported, recurrent administration of nitroglycerin (NTG) can establish a chronic migraine model in rodents through central sensitization. Chronic intermittent intraperitoneal injection of 10 mg/kg NTG every 2 days for 9 days evokes a progressive and sustained basal hypersensitivity, in which animals are sensitive to mechanical stimulation days after NTG administration, consistent with clinical observations of patients with chronic migraine in whom allodynia may occur both between and during migraine attacks (18). The recurrent NTG injection has been widely applied to establish the animal model of chronic migraine in experiments. This study aimed to determine whether the vestibular function was impaired in the recurrent NTG-induced chronic migraine model in rats, and investigate the plasma fibrinogen levels and the effect of defibrinogenation on chronic migraine and associated vestibular dysfunction.

## 2. Materials and methods

### 2.1. Animals

SPF-grade Wistar rats (250–300 g, Charles Rivers) were used for the experiments.

Rats were housed in plastic cages in pairs at 22 ± 2°C with 50 ± 5% humidity on a 12 h light/dark cycle (7 a.m.–7 p.m. lights on), with access to food and water *ad libitum*. All procedures were carried out following the Guide for the Care and Use of Laboratory Animals and approved by the Ethics Committee on Animal Experiments of the Peking University People’s Hospital (2021PHE059).

### 2.2. Drug administration

After a week of adaptive feeding, all rats were randomly divided into the model group (MG, *n* = 12) and blank group (BG, *n* = 6). A stock of 5.0 mg/mL nitroglycerin (NTG, Beijing Yimin Pharmaceutical Co., Ltd., Beijing, China) was freshly diluted to 1 mg/mL in 0.9% saline in a polypropylene tube. The rats in MG were treated with an intraperitoneal injection of 10 mg/kg NTG every 2 days for 9 days to model chronic migraine rats, while those in BG were treated with intraperitoneal 0.9% saline with the same dose as that in MG. The chronic migraine model was considered to be established when completing the last NTG injection on day 9.

Following the modeling, the rats in MG were randomly assigned to the treatment group (TG, *n* = 6) and control group (CG, *n* = 6). Defibrinogenating drug batroxobin (Beijing Tobishi Pharmaceutical Co., Ltd., Beijing, China) was injected intraperitoneally at a dose of 30BU/kg for rats in TG on days 1 and 3 after model establishment, whereas the rats in CG were administrated with the same dose of 0.9% saline.

### 2.3. Behavioral measures

All behavioral measures were conducted by researchers who were blind to treatment conditions.

#### 2.3.1. Mechanical withdrawal threshold

Cutaneous allodynia is a typical symptom of migraine and was measured by assessing the mechanical withdrawal thresholds of rats’ hind paws. Mechanical withdrawal thresholds were determined with von Frey (Semmes-Weinstein) monofilaments (North Coast Medical, Morgan Hill, CA, United States) using the up-and-down method by the instruction of the Up–Down Reader^1^ (19).

The rats were habituated to the testing apparatus for 30 min before the determination of mechanical withdrawal thresholds. In the modeling stage, each group of the rats underwent tests 30 min before and 2 h after injection to obtain the data of baseline and post-treatment mechanical withdrawal thresholds. After model establishment, the rats in TG underwent tests 1 h after each administration of batroxobin.

#### 2.3.2. Assessment of vestibular dysfunction

The vestibular dysfunction was evaluated by negative geotaxis and vestibular dysfunction ratings (VDRs) at 2 h after the fifth NTG or saline injection, as well as 1 h after the last administration of batroxobin.

##### 2.3.2.1. Negative geotaxis

Rats were placed onto a 40° slope (50 × 40 cm w × h) with their head downward, and the mean time to rotate 180° was recorded. The maximum recording time was 20 s (3 trials per rat). The value of all three trials was averaged.

##### 2.3.2.2. Vestibular dysfunction ratings

As previously described, the sensitivity and specificity of VDR in evaluating vestibular deficiency have been confirmed in several experiments in rats (20). In this test, 6 items are rated from 0 (normal behavior) to 4 (highest score of behavioral deficiency) to obtain a total score of 0–24.

Three of these items are alterations in spontaneous behavior that appear in vestibular deficient animals, including stereotyped circling, retropulsion, and head bobbing. Rats were placed individually in a transparent chamber and were recorded for an observation period of 2 min.

The other three are alterations in anti-gravity reflexes: tail-lift reflex, air-righting reflex, and contact inhibition of the righting reflex. For slow-motion analysis, these reflex tests were recorded by high-speed video at 240 frames per second (fps). Each rat was tested 3 times intermittently, and the average value of the 3 trials was documented.

1. Tail-lift reflex

Rats were grasped by the base of the tail and gently lifted to ~40 cm. The minimum angle formed by the nose, the back of the neck, and the base of the tail during the lifting maneuver was measured. This reflex is lost in vestibular deficient rats, which show instead ventral curling, and therefore reduced angles.

2. Air-righting reflex

Rats were held in a supine position at ~40 cm of height and released to fall on a foam cushion. The time from the release of the rat until it fully righted its head was measured. The time taken is longer in vestibular deficient rats.

3. Contact inhibition of the righting reflex

Rats were placed in a supine position on a horizontal plane, and another horizontal surface was placed on their feet to observe whether the rat could turn from the supine position to the right.

### 2.4. Determination of the plasma fibrinogen concentration

**Figure 1:**
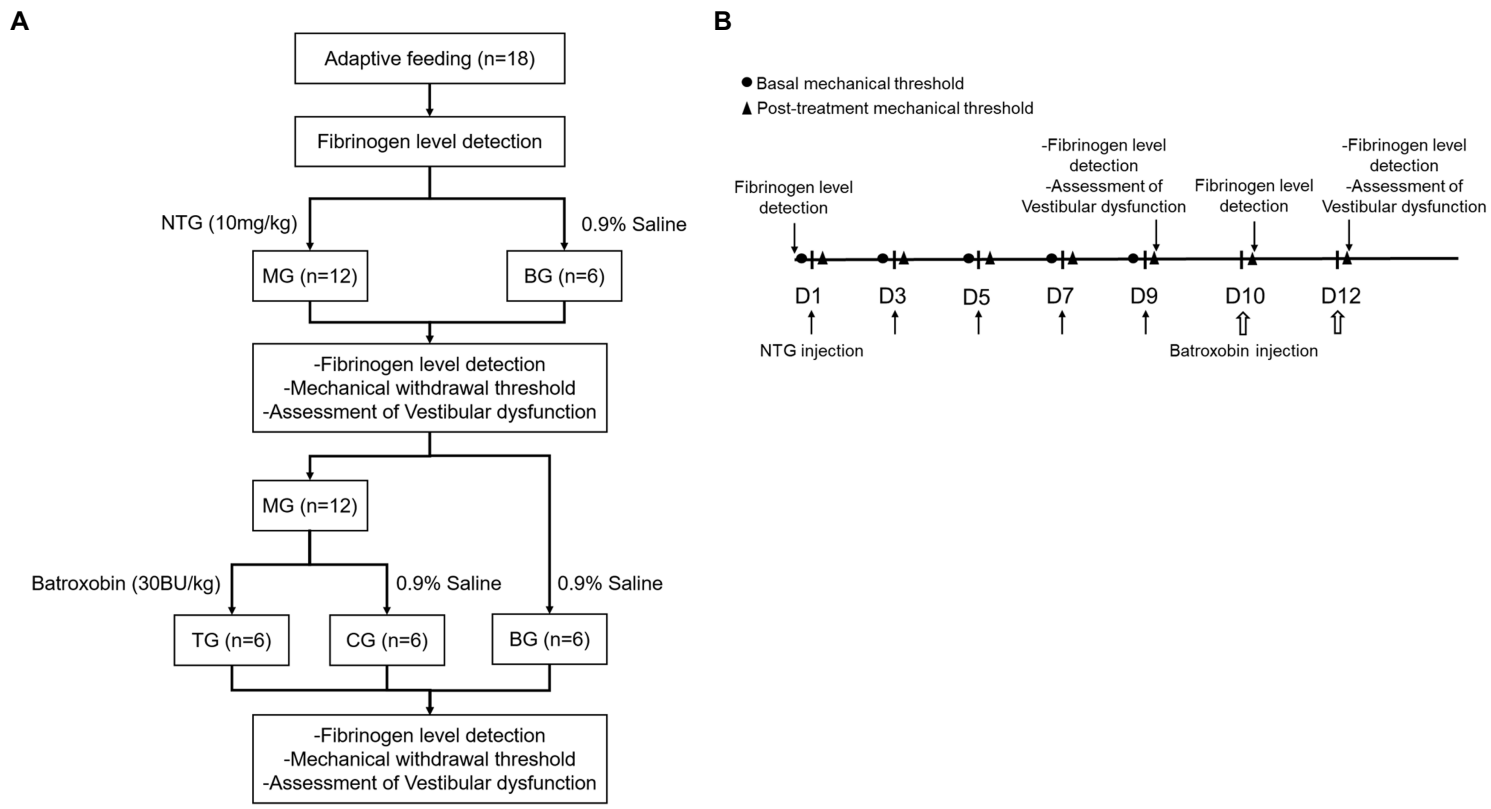
Experimental procedures. **(A)** Flow chart of the experiment, **(B)** Timeline of behavior studies’ protocol.

The enzyme-linked immunosorbent assay (ELISA) kit measures the concentration of fibrinogen in the plasma. Blood samples were collected from the tail vein by EDTA anticoagulant tube before the first NTG injection, 2 h after the fifth NTG injection, and 1 h after each batroxobin injection. Plasma was obtained by centrifuging (3,000 rpm, 20 min) after standing at room temperature. A standard ELISA curve was generated for the 96-well plate. 50 μL of the sample was added to each well while leaving a blank well. 50 μL of HRP conjugate was added to each well and the plate was incubated at 37°C for 30 min. After the incubation, wash the plate 5 times, 50 μL of TMB working solution was added to each well, and the plate was incubated at 37°C for 10 min and was protected from light. Afterward, 50 μL of stop solution was added to each well. The optical density was measured at 450 nm using a microplate reader (Figure 1).

### 2.5. Statistical analyses

All data were presented as mean ± SD. Student’s *t*-test was used for statistical comparisons between the two groups. One-way ANOVA followed by *post hoc* analysis with the Tukey test was used for statistical comparisons among groups. Statistical significance was defined as two-tailed *p* < 0.05. Statistical analyses were performed by SPSS software version 24.0 and GraphPad Prism 7.0.

## 3. Results

### 3.1. Recurrent NTG injection-induced mechanical hyperalgesia

**Figure 2:**
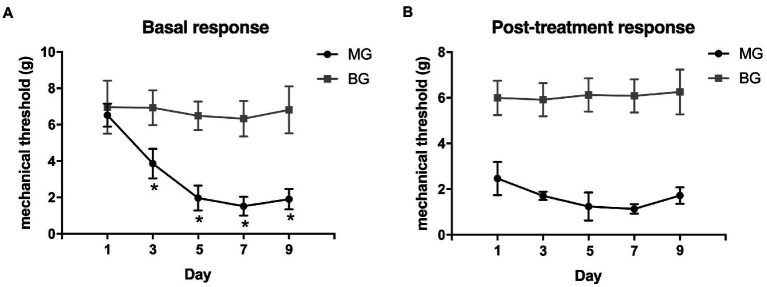
Recurrent NTG injection-induced mechanical hyperalgesia. **(A)** the basal mechanical withdrawal thresholds decreased significantly after NTG injection in a time-dependent manner and **(B)** post-treatment mechanical withdrawal thresholds also decreased significantly after each NTG administration. *p < 0.05.

The changes in mechanical withdrawal thresholds were tested by von Frey monofilaments. Baseline mechanical withdrawal thresholds were decreased progressively in a time-dependent manner, and recurrent NTG injection produced a significant reduction in mechanical withdrawal thresholds on day 3, 5, 7, and 9 (Figure 2A). The post-treatment mechanical withdrawal thresholds of rats in MG after each NTG administration were significantly lower than that in the BG (Figure 2B, *p* < 0.001). These results revealed that a single administration of nitroglycerin induced acute allodynia, and there were both acute and chronic mechanical hyperalgesia during repeated intermittent administration of NTG.

Rats in MG also showed some behavior changes, including frequent head scratching, grooming, and cage climbing, as well as orbital tightening and cheek flattening, which were regarded as positive reactions of migraine (see Supplementary material).

### 3.2. Recurrent NTG injection-induced vestibular dysfunction

**Figure 3:**
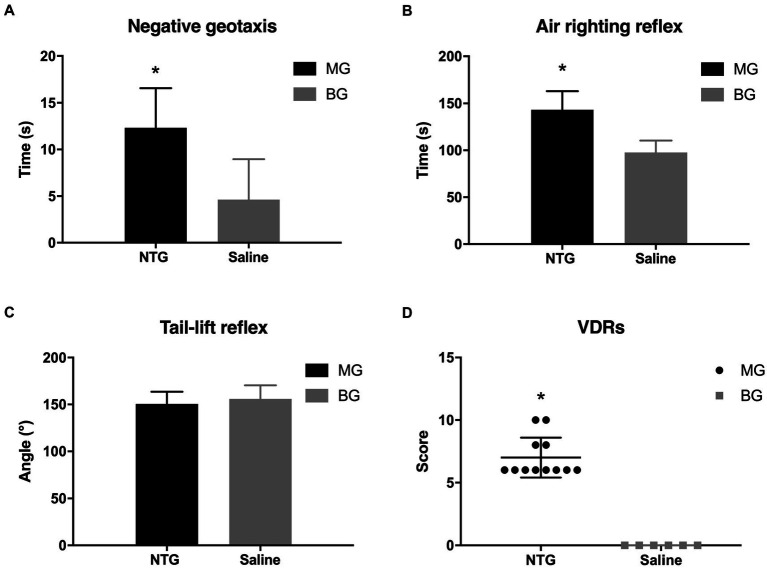
Recurrent NTG injection-induced vestibular dysfunction. Compared with rats in BG, rats in MG spent a significantly longer time in the negative. Geotaxis test **(A)** and the air-righting reflex test **(B)**, as well as scored higher in the VDRs **(D)**. In the tail-lift reflex test **(C)**, however, no significant difference was observed between MG and BG in the angle formed by the tip of the nose, in the back of the neck, and the base of the tail. *p < 0.05.

The alternations of vestibular function were determined by negative geotaxis and vestibular dysfunction ratings (stereotyped circling, retropulsion, head bobbing, tail-lift reflex, air-righting reflex, and contact inhibition of the righting reflex). In the negative geotaxis test (Figure 3A), rats in MG spent approximately triple the time to turn 180° upward as compared to the BG (12.32 ± 4.26 s vs. 4.33 ± 1.63 s, *p* < 0.001). There were 4 (33.3%) rats in MG that showed stereotyped circling, whereas no alterations in spontaneous behavior were observed in BG. In the air righting reflex test (Figure 3B), the time required for the rats in MG to right from the supine position was significantly longer than those in BG (143.28 ± 19.78 ms vs. 97.19 ± 12.61 ms, *p* < 0.001). In the tail-lift reflex test (Figure 3C), the angle formed by the tip of the nose, the back of the neck, and the base of the tail in MG was slightly reduced, but there was no significant difference as compared with BG (150.61 ± 13.03° vs. 155.97 ± 14.51°, *p* = 0.357). The VDRs of the rats in MG were significantly higher than those in BG (7.00 ± 1.59 vs. 0, *p* = 0.001, Figure 3D). Behavior studies showed that recurrent injection of NTG induced vestibular dysfunction in rats.

### 3.3. Recurrent NTG injection induced elevated fibrinogen level

**Figure 4:**
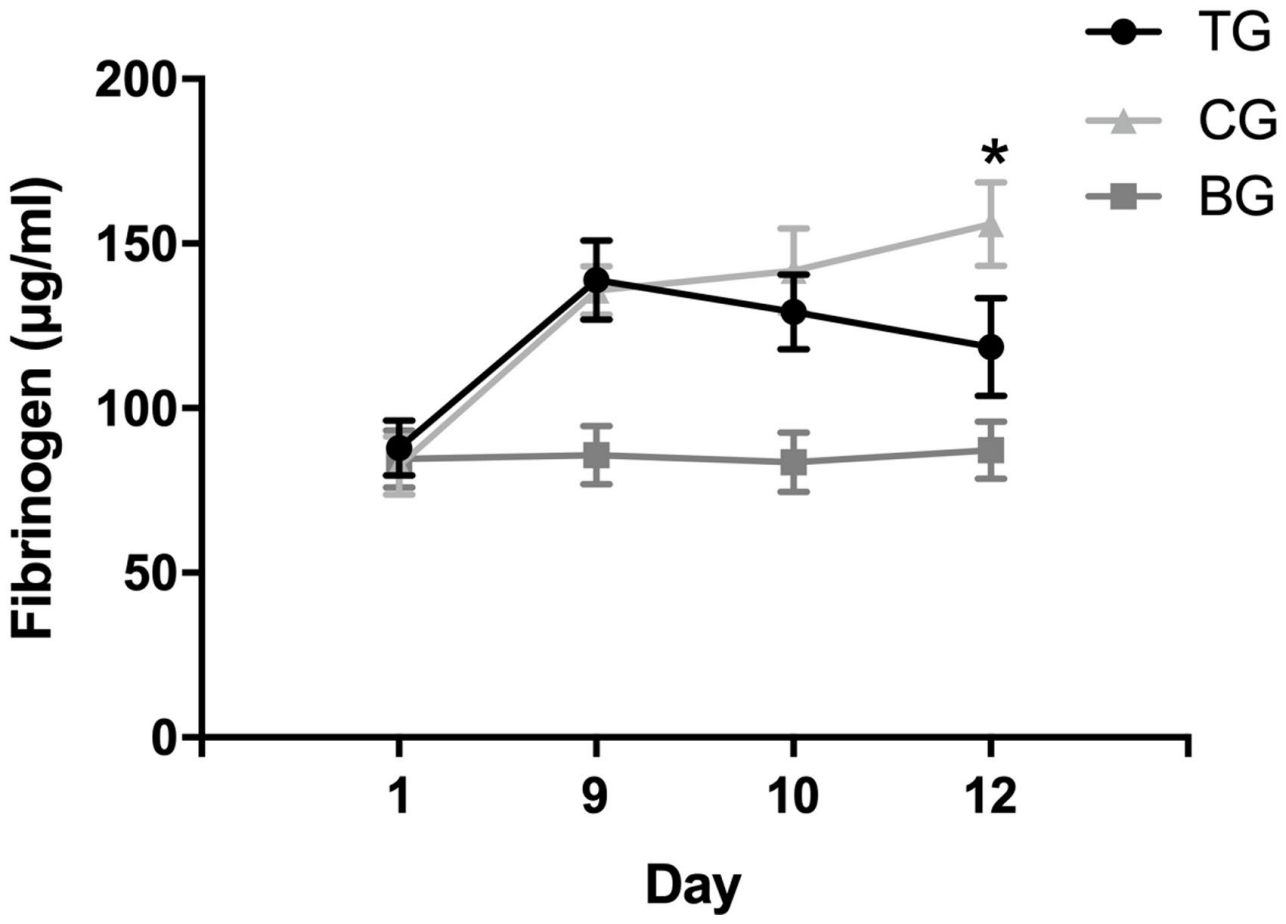
Plasma fibrinogen levels after NTG and batroxobin administration. On day 9, the levels of plasma fibrinogen in model rats (TG and CG) were significantly higher than those in BG after recurrent NTG injection, suggesting that recurrent NTG injection increased the levels of plasma fibrinogen. After the batroxobin administration, plasma fibrinogen levels in TG decreased and were significantly lower than CG on day 12, suggesting that two treatments of batroxobin reduced plasma fibrinogen levels in model rats significantly. *p < 0.05.

The effect of recurrent NTG injection on plasma fibrinogen levels was evaluated by ELISA analysis. The plasma fibrinogen in MG was elevated after modeling and was significantly higher than that in BG (137.88 ± 11.05 vs. 86.19 ± 8.48, *p* < 0.01). Recurrent NTG injection increased model rats’ plasma fibrinogen levels (Figure 4).

### 3.4. Defibrinogenating effect of batroxobin

As shown in Figure 4, no significant statistical difference was observed in plasma fibrinogen levels between TG and CG before the batroxobin administration (87.97 ± 8.47 μg/mL vs. 82.63 ± 8.91 μg/mL, *p* = 0.718). The level of plasma fibrinogen in TG decreased after the first administration of batroxobin, however, there was no statistically significant difference compared with the level in CG (129.31 ± 11.32 μg/mL vs. 141.76 ± 12.82 μg/mL, *p* = 0.233). After the second administration of batroxobin, the level of plasma fibrinogen in TG further decreased to near levels in BG and was significantly lower than that in CG (155.97 ± 12.68 μg/mL vs. 118.60 ± 14.84 μg/mL, *p* = 0.008). Compared with CG, batroxobin could significantly reduce plasma fibrinogen levels in model rats after two treatments.

### 3.5. Defibrinogenation had no effect on the mechanical hyperalgesia and vestibular dysfunction

**Figure 5:**
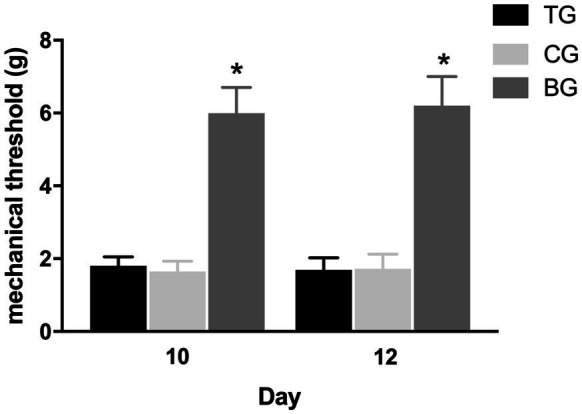
Defibrinogenation had no effect on the mechanical hyperalgesia. The mechanical withdrawal threshold in TG following each batroxobin administration on day 10 and 12 did not differ significantly from CG. *p < 0.05.

**Figure 6:**
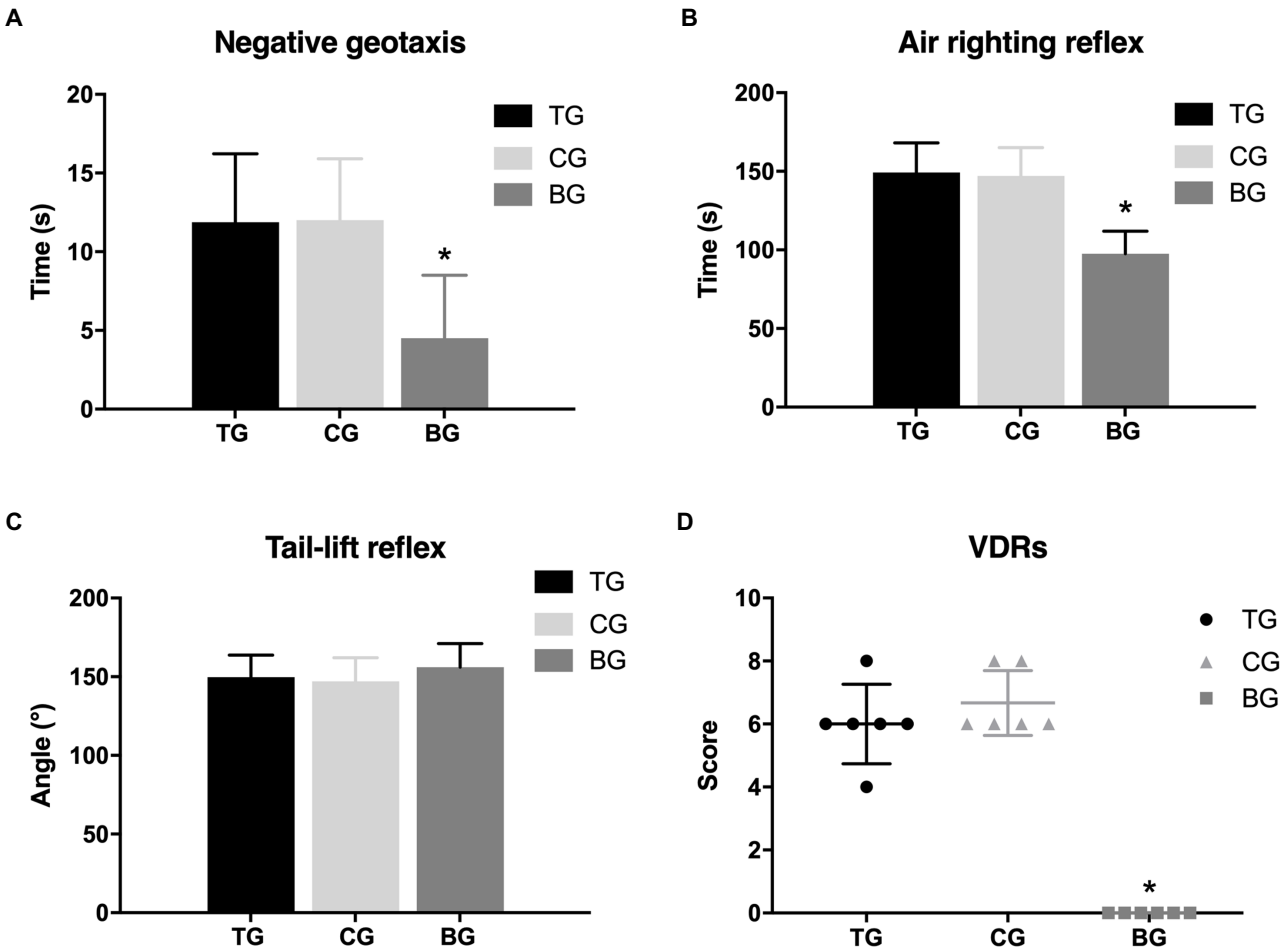
Defibrinogenation did not affect vestibular dysfunction. There was no significant difference between TG and CG in the negative geotaxis **(A)**, air-righting reflex test **(B)**, and tail-lift reflex **(C)**, as well as the VDRs **(D)**. *p < 0.05.

Figure 5 showed that the mechanical withdrawal threshold in TG following each batroxobin administration did not differ significantly from CG. And compared with BG, mechanical hyperalgesia was still present. Similarly, it can be seen from Figure 6 that the performance of negative geotaxis, tail-lift reflex, and air-righting reflex did not improve in TG. There was also no significant difference in VDRs between TG and CG, which both performed significantly worse than BG.

## 4. Discussion

In this study, vestibular dysfunction was observed in the NTG-induced chronic migraine rat models, and the levels of plasma fibrinogen were increased following recurrent NTG injection. However, defibrinogenation had no effect on mechanical hyperalgesia and vestibular dysfunction in the chronic migraine model rats.

Studies in patients with vestibular migraine have reported that the onset of vertigo attacks occurred on average 9 years later than the onset of migraine headaches (21). And the prevalence of vertigo prominently increased in chronic migraine compared with episodic migraine (22). These findings indicate that animal models of chronic migraine are more likely to develop vestibular dysfunctions. Recurrent administration of NTG has been validated to be a reliable method for establishing an animal model of chronic migraine (23). In this study, rats in MG exhibited acute and chronic persistent mechanical hyperalgesia, as well as other associated symptoms such as facial grimace and behavioral changes, suggesting that the rat model of chronic migraine was successfully established. The performance of the behavior test for assessing vestibular function in MG was significantly worse than those in BG, supporting that the NTG-induced model rats developed vestibular dysfunction. These findings suggest that the NTG-induced chronic migraine rat model can be used for experimental research on migraine-associated vestibular symptoms. Consistent with our results, Zhang et al. reported that the development of vestibular dysfunction coincides with that of hyperalgesia in the rat model of chronic migraine induced by NTG (24), and the expression of c-Fos and calcitonin gene-related peptide was significantly increased in the vestibular nucleus of model rats, suggesting that sensitization of the vestibular nucleus may impair vestibular function in chronic migraine. The abnormalities in the vestibular pathway can be further explored using chronic migraine model induced by recurrent NTG administration in rats to clarify the mechanism of vestibular dysfunction in migraine.

In this study, it was found that after recurrent injection of NTG, the levels of plasma fibrinogen in rats increased, which were consistent with findings in patients with migraine. A population-based study found that plasma fibrinogen levels were significantly higher in migraine patients compared with controls and were more closely associated with migraine with aura (14). Fibrinogen levels in the blood-cerebrospinal fluid were also increased in patients with migraine (25). There may be several reasons for the increase in fibrinogen. First, hypercoagulability is closely related to migraine, and fibrinogen participated in the coagulation process. Villiers et al. (26) investigated the structure and polymerization kinetics of fibrinogen in patients with migraine using scanning electron microscopy and fluorescent microscopy, reporting that the tertiary structure of fibrinogen was altered from mainly α-helices to mainly β-sheets. The alterations contribute to abnormal hypercoagulability and increase the risk of microembolization. Second, the coagulation system overlaps with the immune system. Increased plasma fibrinogen levels have been considered not only part of the hemostatic system activation, but also part of the acute inflammatory response. In the NTG-induced migraine model, NTG mainly acts as a NO donor to induce migraine by activating the trigeminal nerve vascular system (27). The modeling mechanism is that NO stimulates soluble guanylate cyclase in cells to generate the second messenger cyclic guanosine monophosphate, thereby activating protein Kinase G, causing Ca^2+^ reuptake and the opening of Ca^2+^ − activated potassium channels, which leads to the vasodilation and plasma protein extravasation, resulting in neurological inflammation (28), and then increased the levels of fibrinogen. Elevated fibrinogen levels, in turn, can induce hypercoagulable inflammatory state and endothelial injury (29). Recent research has uncovered important roles for fibrinogen in the activation of neuroinflammation (30). Therefore, high plasma fibrinogen levels may be the result of migraine attacks, and may also be a contributor to the persistence and exacerbation of symptoms. Moreover, the hemostatic system is also involved in acute peripheral vestibular dysfunction (17, 31). Milionis et al. reported that in patients with vestibular neuritis, plasma fibrinogen levels were significantly higher during the acute episode than in the stable state (32). Both clinical and preclinical studies in rats found positive associations between mental stress and plasma fibrinogen concentration (33, 34). Migraine attacks and acute vestibular disorders both trigger acute stress, which may be another pathway resulting in elevated plasma fibrinogen levels.

It was speculated that fibrinogen may serve as a therapeutic target for neurological diseases, and multiple animal experiments have confirmed the protective and therapeutic effects of the depletion of fibrinogen on neurological inflammation (30). A nationwide multi-center study on sudden hearing loss in China reported that defibrinogenation therapy gave better relief of headaches in patients suffering from total frequency hearing loss accompanied by headache (35). To determine whether defibrinogenation affected mechanical hyperalgesia and vestibular dysfunction in the migraine model rats, batroxobin was administrated after model establishment. Batroxobin is a defibrinogenating drug that showed high affinity with fibrinogen, produced from the venom of *Bothrops atrox moojeni* (36). In this study, batroxobin lowered the fibrinogen levels in TG and led to a significant decrease compared with CG following the second administration of batroxobin. Nevertheless, there was no significant improvement in mechanical hyperalgesia and vestibular function after the fibrinogen levels were lowered. There are several possible reasons for this observation. First, there may be different outcomes based on different animal models. A chronic migraine model was used in this study, and defibrinogenation may not be sufficient to improve hyperalgesia and vestibular dysfunction after recurrent migraine attacks. Thus, additional studies on animal models of acute migraine should be further investigated. Second, the dose of batroxobin (30BU/kg) was referred to the dose used in the study on multiple sclerosis and demyelinating diseases (37), which may not be suitable for the treatment of chronic migraine. Other doses should be tested in this model to further describe the effect. Third, the side effect of the drug may interfere with the results, as headache and dizziness were reported to be adverse reactions of batroxobin.

The present study has some limitations. First, the assessment of vestibular function was entirely based on the behavioral test, and the results were determined according to the subjective judgment of researchers. Vestibular function tests such as vestibular evoked potentials and electronystagmography can increase the reliability of the results. Second, the study did not carry out a histological evaluation in the peripheral vestibular organs to support vestibular dysfunction. Third, other migraine-related molecular markers were not included in the study. c-Fos is an indicator for neuronal activation by noxious stimuli and is widely used in the study of the mechanism of migraine (38). In the future study, we will investigate the pathologic changes in the peripheral vestibular organs, and the changes in c-fos expression after defibrinogenation should be detected to further elucidate the association between fibrinogen and migraine. In addition, the changes in the level of fibrinogen in acute migraine models should also be explored.

## Data availability statement

The raw data supporting the conclusions of this article will be made available by the authors, without undue reservation.

## Ethics statement

The animal study was reviewed and approved by Ethics Committee on Animal Experiments of the Peking University People’s Hospital.

## Author contributions

XM, LY, and TD: concept and design. JZ, YZ, YJ, LH, XM, LY, and TD: acquisition, analysis, or interpretation of data. JZ and YZ: drafting and refining of the manuscript. YJ and LH: critical revision of the manuscript for important intellectual content. All authors contributed to the article and approved the submitted version.

## Funding

This work was supported by Peking University People’s Hospital Scientific Research Development Funds RDL2021-14, RDY2021-25, and 2021-Z-56.

## Conflict of interest

The authors declare that the research was conducted in the absence of any commercial or financial relationships that could be construed as a potential conflict of interest.

## Publisher’s note

All claims expressed in this article are solely those of the authors and do not necessarily represent those of their affiliated organizations, or those of the publisher, the editors and the reviewers. Any product that may be evaluated in this article, or claim that may be made by its manufacturer, is not guaranteed or endorsed by the publisher.

## Footnotes

^1^ https://sourceforge.net/projects/updownreader/
