# Supplementary figures and images for: The plasma fibrinogen levels in the nitroglycerin-induced chronic migraine rat model and its association between migraine-associated vestibular dysfunction
Source: Front Neurol. 2023 Mar 24;14:980543. doi: 10.3389/fneur.2023.980543 (PMC10079898; doi:10.3389/fneur.2023.980543)

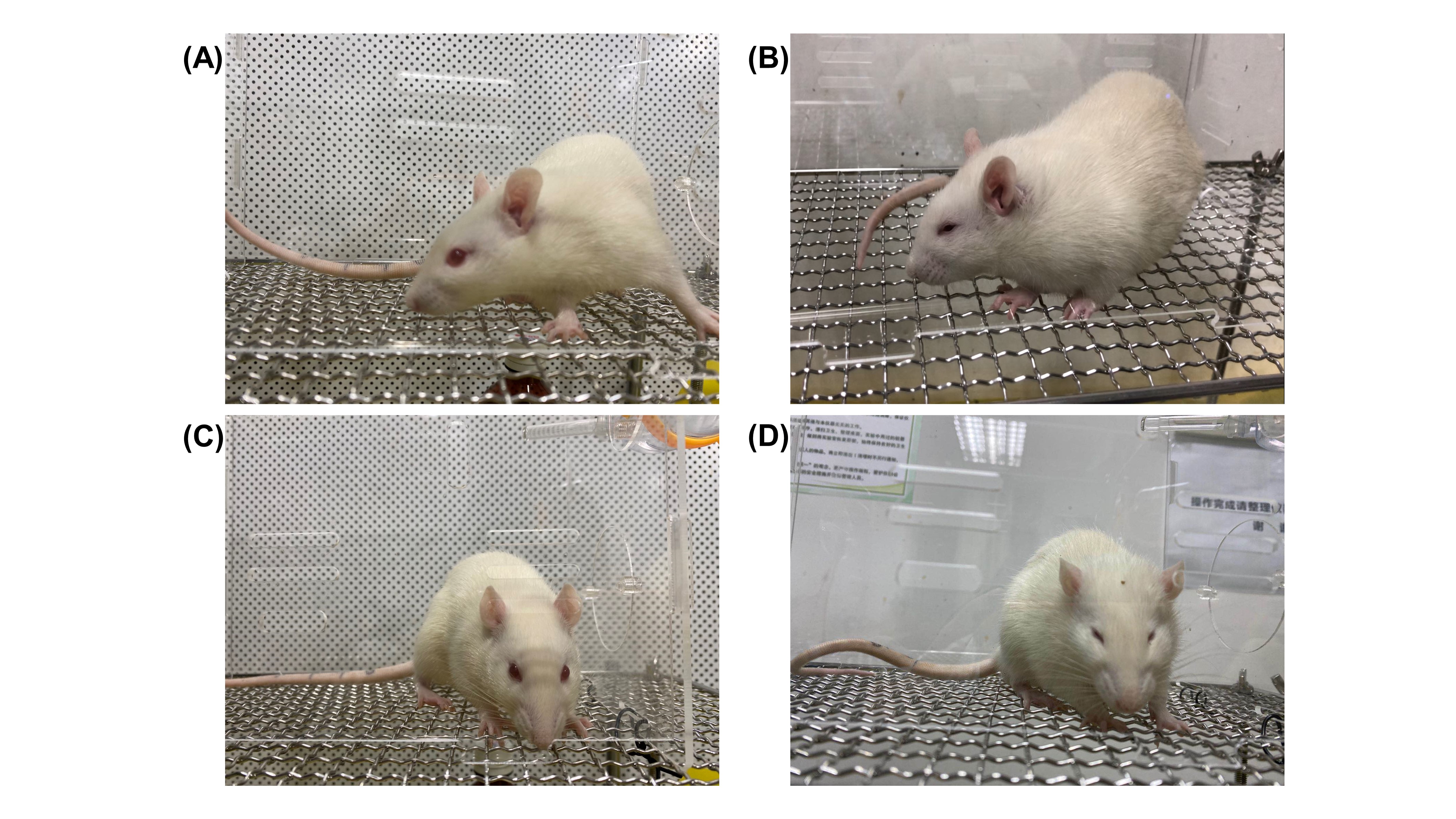

Supplement: Supplementary file 1 [file Image_1.JPEG]
